# Prevalence and predictors of being lost to follow-up after transurethral resection of the prostate

**DOI:** 10.1038/s41598-018-24869-z

**Published:** 2018-04-23

**Authors:** Matteo Fontana, Luca Boeri, Andrea Gallioli, Elisa De Lorenzis, Franco Palmisano, Stefano Paolo Zanetti, Gianluca Sampogna, Giancarlo Albo, Fabrizio Longo, Franco Gadda, Paolo Guido Dell’Orto, Emanuele Montanari

**Affiliations:** IRCCS Fondazione Ca’ Granda, Ospedale Maggiore Policlinico, Department of Urology, University of Milan, Milan, Italy

## Abstract

Patient follow-up after transurethral resection of the prostate (TURP) is crucial to evaluate treatment-related outcomes and potential adverse events. We sought to determine the rate of, and factors associated with, patient nonadherence to follow-up after TURP. Data from 180 patients who underwent TURP were analysed. Patient counselling and follow-up were standardized among the cohort. Patients were considered lost to follow-up (LTF) if they were at least 30 days from their first scheduled follow-up appointment. Descriptive statistics and logistic regression analyses were performed to determine the impact of predictors on the rate of compliance with prescribed follow-up. Of 180 patients, 55 (30.5%) were LTF. LTF patients were younger (p < 0.001), had lower educational status (p = 0.007) and were more frequently single (p = 0.03) than those who were not LTF. Importantly, patients who experienced a postoperative-related event (PRE) were more likely to follow-up (p = 0.04). Multivariable analysis revealed that younger age (p < 0.001) and low educational status (p < 0.001) were independent predictors of being LTF. One out of three men submitted to TURP is lost to follow-up in the real-life setting. Noncompliance to follow-up was more frequent among young, single patients with low educational status. On the contrary, patients who experienced a PRE were more likely to follow-up.

## Introduction

Benign prostatic hyperplasia (BPH) is a common cause of lower urinary tract symptoms (LUTS) in adult and elderly males and has a significant impact on a patient’s quality of life (QoL)^1^. Since BPH is a chronic condition associated with ageing, its economic burden is remarkable and likely to increase with future demographic changes^2^.

Trans-urethral resection of the prostate (TURP) is widely recognized as an effective endoscopic surgical technique for the treatment of patients with LUTS due to BPH (LUTS/BPH) resistant to medical therapy^1–3^. Although innovative techniques have been developed in recent years, TURP still represents the gold standard for the treatment of LUTS/BPH, being performed worldwide as the technique of choice by thousands of Urologists.

Despite being considered an effective and safe technique, TURP may be associated with various short-term and long-term complications^4^. Temporary difficult micturition and acute urinary retention, persistent macrohematuria and lower urinary tract infections are the most common short-term complications after TURP. Long-term complications, such as retrograde ejaculation, bladder neck contracture, urethral strictures and incontinence may also appear over the course of many years following surgery, potentially compromising functional outcomes and possibly leading to the need for further treatment^5^.

An accurate follow-up after TURP is important to assess, and eventually treat, potential surgical complications, to estimate the success of the procedure and to evaluate patient satisfaction after surgery. In this context, patient collaboration is fundamental: nonetheless, many patients do not attend follow-up appointments and are consequently lost to follow up (LTF).

Non-adherence of patients to the prescribed therapeutic regimens and follow-up visits has become a critical issue in recent years and has been thoroughly investigated in chronic pathologies such as diabetes or hypertension^6,7^. However, the current literature regarding patient follow-up after surgical procedures is ambiguous and of difficult interpretation because, on one hand, retrospective studies examining outcomes of procedures or therapeutic regimens necessarily exclude patients without available follow-up visits and, on the other hand, prospective studies generally report optimal follow-up within their protocols.

Non-adherence to follow up visits has a significant impact in the field of urology as well. Despite the risk that evading follow up visits could compromise clinical outcomes and potentially lead to serious events, such as the misdiagnosis of treatment-related complications or other associated pathologies, the reasons associated with being LTF in the field of urology have been scantly investigated in the real-life setting^8–10^. This is especially true for patients submitted to TURP for LUTS/BPH.

To try to address this gap we performed a cross-sectional study assessing the adherence of patients treated with TURP to follow-up visits in a real-life setting, attempting to identify potential predictive factors of patients who are at higher risk of evading medical attention, with the goal of improving counselling for these patients.

## Results

The initial cohort of patients included 188 men submitted to TURP, however, 6 patients were definitively excluded from the analysis for missing data. We also decided to exclude 2 patients who had private insurance. Given the low percentage of patients in Italy who access health care through private insurance, we believe that excluding these 2 patients left us with a more representative sample of the Italian population. Of 180 patients who underwent TURP, 55 (30.5%) were LTF.

Table 1 reports demographics characteristics of the whole cohort of patients. LTF patients were younger (65.8 vs. 71.6 yrs; p < 0.001), had a lower educational status (p = 0.007) and were more frequently single (p = 0.03) compared to those who were not LTF. On the contrary, no differences in terms of BMI, CCI, rate of psychiatric disorders, distance travelled and preoperative catheterization were seen between groups. Similarly, preoperative PSA, PV and Qmax did not affect LTF rate (Table 1).

**Table 1:** Baseline preoperative characteristics and descriptive statistics of participants (No. = 180; mean (SD), [range]).

	Overall	LTF	−LTF	p-value (F)*
No. of patients (%)	180 (100)	55 (30.5)	125 (69.5)	
Age (years)	69.8 (8.5)	65.8 (9.4)	71.6 (7.4)	<0.001 (16.4)
	[48–87]	[48–87]	[55–87]	
BMI [kg/m^2^]	26.1 (4.1)	25.8 (3.5)	26.3 (4.3)	0.55 (0.35)
	[18.4–42.9]	[19.5–33.6]	[18.4–42.9]	
CCI categorized [No. (%)]				0.73 (*X*_2_ = 0.11)
0	75 (41.6)	24 (43.5)	51 (40.6)	
≥1	105 (58.4)	31 (56.5)	74 (59.4)	
Educational Status [No. (%)]				0.007 (*X*_2_= 7.2)
Primary/ Secondary school	82 (45.5)	34 (61.7)	48 (38.4)	
High school/University	98 (54.5)	21 (38.3)	77 (61.6)	
Marital Status [No. (%)]				0.038 (*X*_2_ = 4.30)
Single	29 (16.2)	14 (25.5)	15 (12.1)	
Married	151 (83.8)	41 (74.5)	110 (87.9)	
Psychiatric disorders [No. (%)]	4 (2.2)	1 (1.8)	3 (2.4)	0.32 (*X*_2_ = 1.01)
Distance traveled (km)	23.5 (77.7)	34.1 (98.3)	19.9 (51.2)	0.26 (1.23)
	[0.3–603]	[0.7–603]	[0.3–465]	
POC [No. (%)]	62 (34.4)	22 (40.4)	40 (31.8)	0.29 (*X*_2_ = 1.08)
Time of POC (months)	8.7 (8.8)	7.7 (3.0)	9.3 (10.9)	0.53 (0.39)
	[2–60]	[2–14]	[3–60]	
Total IPSS score	17.6 (4.4)	17.3 (5.5)	17.9 (5.1)	0.21 (1.51)
	[12–33]	[12–33]	[12–31]	
PSA (ng/ml)	4.2 (3.8)	3.5 (2.5)	4.4 (4.3)	0.17 (1.89)
	[0.1–8.4]	[0.3–5.2]	[0.1–8.4]	
Prostate Volume (ml)	79.6 (45.7)	71.1 (35.5)	83.1 (49.0)	0.15 (2.09)
	[14–160]	[14–141]	[15–160]	
Flow Max (ml/sec)	13.9 (19.1)	16.1 (25.4)	12.7 (14.8)	0.44 (0.58)
	[2.1–52.0]	[2.1–52.0]	[2.1–42.0]	

Keys: LTF = Lost to follow up; BMI = body mass index; CCI = Charlson Comorbidity Index; POC = Preoperative catheterization; PSA = Prostate Specific Antigen; IPSS = International Prostatic Symptoms Score.

^*^P value according to chi-square test or analysis of variance (ANOVA), as indicated.

With regard to perioperative outcomes, patients who experienced a postoperative-related event were more likely to follow-up (30.2% vs. 14.9%; p = 0.04) than those who missed their scheduled visit. Similarly, patients with a higher Clavien-Dindo complication score were more frequently not LTF (p = 0.03). Surgery time, Haemoglobin values, catheterization time and length of stay did not statistically differ between groups (Table 2).

**Table 2:** Baseline intraoperative and postoperative descriptive statistics of participants (No. = 180; mean (SD), [range]).

	Overall	LTF (N=55)	−LTF (N=125)	p-value (F)*
Preoperative Hb (g/dl)	14.2 (1.4)	14.4 (1.5)	14.0 (1.3)	0.22 (1.47)
	[9.8–17]	[9.8–16.6]	[10–17]	
Surgery time (min)	105.5 (46.9)	98.7 (40.8)	108.5 (48.9)	0.23 (1.44)
	[20–320]	[30–190]	[20–320]	
Resected tissue (g)	57.1 (42.2)	53.8 (36.4)	58.8 (44.3)	0.44 (0.58)
	[10.0–180]	[10.0–150]	[10.0–180]	
Postoperative Hb (g/dl)	12.4 (1.6)	12.4 (1.6)	12.3 (1.6)	0.81 (0.05)
	[8.8–16.3]	[9.3–16.3]	[8.8–16.1]	
Catheterization time (days)	3.2 (1.5)	3.1 (1.2)	3.2 (1.7)	0.53 (0.39)
	[1–14]	[2–8]	[1–14]	
Length of stay (days)	4.9 (2.6)	4.6 (1.4)	5.0 (3.0)	0.49 (0.46)
	[2–29]	[3–9]	[2–29]	
PRE [No. (%)]				0.04 (*X*_2_ = 4.01)
No	134 (74.5)	47 (85.1)	87 (69.8)	
Yes	46 (25.5)	8 (14.9)	38 (30.2)	
Clavien-Dindo [No. (%)]				0.03 (*X*_2_ = 7.28)
0	137 (76.1)	48 (87.2)	89 (71.0)	
1	23 (12.7)	1 (2.1)	22 (17.8)	
≥2	20 (11.1)	6 (10.6)	14 (11.2)	
Total IPSS score			5.2 (1.5)	
			[1–25]	
Flow Max (ml/sec)			26.6 (11.4)	
			[7.1–48.0]	

Keys: LTF = Lost to follow up; Hb = Haemoglobin values; PRE = postoperative-related events; IPSS = International Prostatic Symptoms Score.

^*^P value according to chi-square test or analysis of variance (ANOVA), as indicated.

The primary reasons for being LTF were “feeling healed”, “concomitant health diseases”, “costs” and “forgot the visit” in 32 (58%), 3 (5%), 8 (14%) and 13 (23%) patients, respectively.

Table 3 reports UVA evaluating the potential associations between demographic and perioperative characteristics and LTF status. Younger age (p < 0.001), lower educational status (p = 0.008) and being single (p = 0.04) were statistically associated with LTF status. In addition, neither comorbidity, distance travelled, nor perioperative clinical parameters predicted follow-up. Only PRE was significantly associated with not being LTF.

**Table 3:** Univariable analysis evaluating potential association between demographic and perioperative characteristics and tendency to following up in the whole cohort.

	Odds ratio	95% CI	p-value
Age (years)	0.91	0.87–0.96	<0.001
BMI [kg/m^2^]	0.97	0.88–1.07	0.55
CCI categorized			
0	Reference		
≥1	1.32	0.44–1.78	0.738
Educational Status			
Primary/ Secondary school	Reference		
High school/University	0.38	0.19–0.78	0.008
Marital Status			
Single	Reference		
Married	0.40	0.16–0.96	0.042
Distance traveled (km)	1.12	0.99–1.1	0.31
POC	1.46	0.71–2.96	0.299
PSA (ng/ml)	0.93	0.84–1.03	0.175
Prostate Volume (ml)	0.99	0.98–1.02	0.152
Flow Max (ml/sec)	1.01	0.98–1.03	0.43
Surgery time (min)	0.99	0.98–1.03	0.232
Catheterization time (days)	0.92	0.72–1.18	0.529
Length of stay (days) PRE	0.94	0.81–1.11	0.502
No	Reference		
Yes	0.41	0.16–0.94	0.048
Clavien-Dindo [No. (%)]			
0	Ref.		
1	0.72	0.25–0.74	0.026
≥2	0.84	0.34–0.85	0.046

Keys: BMI = body mass index; CCI = Charlson Comorbidity Index; POC = Preoperative catheterization; PSA = Prostate Specific Antigen; PRE = postoperative-related events.

Table 4 shows MVA analyses assessing potential predictors of LTF in the whole cohort. Younger age (OR 0.89, p < 0.001) and low educational status (OR 0.23, p < 0.001) were independent predictors of being LTF, after accounting for CCI, marital status, distance travelled and the presence of PREs.

**Table 4:** Logistic regression models predicting being LTF in the whole cohort.

	Odds ratio	95% CI	p-value
Age (years)	0.89	0.84–0.95	<0.001
CCI categorized			
0	Reference		
≥1	1.12	0.50–2.51	0.776
Educational Status			
Primary/ Secondary school	Reference		
High school/University	0.23	0.09–0.55	0.001
Marital Status			
Single	Reference		
Married	0.45	0.16–1.28	0.137
Distance traveled (km)	1.16	0.99–1.1	0.88
PRE			
No	Reference		
Yes	0.55	0.19–1.59	0.27

Keys: CCI = Charlson Comorbidity Index; PRE = postoperative-related events.

## Discussion

The aim of our study was to assess the prevalence of patients undergoing TURP for LUTS/BPH that miss their follow up visit (namely, lost to follow up) and to investigate potential clinical predictors of LTF status in a real-life setting. From the experience gathered over the course of more than two years, we showed that one out of three men is LTF after TURP. Moreover, we found that LTF men were younger, had a lower educational status and were more frequently single than those who were not LTF. Interestingly, we also noted that patients who experienced a postoperative-related event and those with a higher Clavien-Dindo complication score were more likely to follow-up after TURP.

Patient adherence to physician prescriptions is nowadays an important issue in medicine, and the development and diffusion of patient-centered approaches has given further relevance to this matter^11–13^. Previous literature on this topic has only focused on chronic medical conditions, such as type 2 diabetes, chronic hepatitis, psychiatric disorders and HIV infection. However, the importance of patient adherence to prescribed therapy and follow up visits has become increasingly evident in the field of urology as well^8^.

To the best of our current knowledge, we conducted the first study examining the rate and predictors of being LTF in patients surgically treated for LUTS/BPH in the real-life setting. In fact, the currently available literature focusing on the follow-up of surgical patients is ambiguous and of difficult interpretation since retrospective studies usually exclude patients without available follow-up visits and prospective studies obviously report optimal follow-up within their protocols, thus shadowing the reality of the everyday clinical practice.

LTF rates in the field of urology were first investigated in patients surgically treated for urolithiasis, where a LTF status of 23% and 18.5% was reported for patients undergoing either ureteroscopy or percutaneous nephrolithotomy, respectively^9,10^. Moreover, both studies took patient factors into account as potential predictors of LTF status, but only Brooks *et al*. considered peri- and post-operative factors in association with being LTF^10^. In particular, Moses *et al*., found that the only LTF-associated factor was government-assisted insurance, independently of age or educational status^9^. On the contrary, Brooks *et al*. reported a trend toward poor compliance among younger patients who traveled a greater distance, but didn’t suggest a correlation with marital status^10^.

In our study we analyzed both patient factors and procedure-related factors (pre-operative and post-operative) in relationship with LTF status. We showed that younger age, lower education status and being single were associated with being LTF. Our findings are in line with the results of previously published studies in the psychological literature showing that the increased LTF rates between single and younger patients with lower educational status were associated with social and emotional instability, employment pressure, financial difficulties and health illiteracy^14,15^. In accordance with Brooks *et al*., we also found that patients who experienced a postoperative-related event or who had a higher Clavien-Dindo score are less frequently LTF, likely because of the fear of other procedure-related consequences.

Of note, previous studies focusing on the LTF rate in urology have failed to identify potential reasons for not attending the prescribed follow up visits. On the contrary, we carefully investigated the reasons of being LTF in our cohort of men surgically treated for LUTS/BPH by directly interviewing patients. The main reasons for LTF status were “feeling healed” after the procedure and forgetfulness in 58% and 23% of cases, respectively. Similar causes of LTF status have been reported by previous studies not specifically related to the urological field, thus highlighting the need for a greater focus on patient counseling regarding adherence to follow up assessments^7–16^. In particular, we can speculate that in highly successful procedures such as TURP, a patient’s belief of being completely healed after the procedure represents a major pitfall for the appropriate follow up of these patients, and should be unquestionably addressed in pre-procedure counselling.

Follow up examinations and visits serve as invaluable instruments to evaluate procedure outcomes and long-term surgical complications, to assess patient QoL and to diagnose associated pathologies. In a cohort of patients surgically treated for LUTS/BPH being LTF clearly puts patients at risk of potentially missing an early diagnoses of prostate cancer due to the interruption of PSA screenings and urological examinations, and of a gradual decline in micturition and QoL due to undiagnosed long-term procedure-related events, which may occur in about 2–10% of these patients^4^.

In order to improve lifelong outcomes and avoid misdiagnosis of long-term complications after surgery, clinicians should make every effort to improve patient follow-up attendance by adopting simple sociological strategies^17^. For example, comprehensive and procedure-focused pre-operative counseling concentrating on the importance of post-procedure follow up assessment and the development of a strong and durable therapeutic relationship between the physician and patient could all help reduce LTF rates^17^.

A major strength of this study is the rigorous methodology and a comprehensive evaluation of both patient and procedure-related factors associated with LTF status, as compared to previous studies that only considered patient–related factors. Another strength is the investigation of the patients’ reported reasons for being LTF, which again highlights the importance of the real-life nature of this study and could lead to the identification of patients who would most benefit from counseling focusing on the importance of post-surgical follow up visits.

Our work is not devoid of limitations. The present study was a cross-sectional retrospective analysis of data stored prospectively: therefore, these findings deserve external validation with an independent, larger and more diverse sample. Although our cohort of patients did not undergo surgery and were not followed by a single Urologist, we standardized pre-operative counseling and post-operative follow up appointments. Nevertheless, variability in terms of communicative skills and attitudes between Urologists may have had an impact on patient adherence to follow up appointments. Additionally, we did not have access to certain variables that could have influenced LTF rates, such as family history of LUTS/BPH and the number of female partners or frequency of sexual intercourse after surgery. It would be useful for future studies to explore the effects of these factors on LTF status. Finally, our results should be interpreted with caution as the limited sample size does not allow for definitive conclusions. However, we believe these findings are clinically relevant because of their strong characterization in the context of the real-life setting. Moreover, further studies may be necessary to define what types of interventions would be most useful for improving follow-up adherence in patients at a greater risk of being LTF.

In conclusion, one out of three men surgically treated per LUTS/BPH is lost at follow up in the real-life setting. Patients who missed their follow up visit were younger, had a lower educational status and were more frequently single than those who were not LTF. Patients who experienced a postoperative-related event and those with higher Clavien-Dindo complication scores were more likely to follow-up after TURP. These findings not only highlight that fact that non-compliance is commonplace in the urologic field of benign prostatic surgery, but also provide useful indications regarding patients who could most benefit from individualized counseling in order to improve follow up adherence.

## Methods

Data from 180 consecutive Caucasian – European patients who underwent TURP for LUTS/BPH at a tertiary referral center from January 2015 to April 2017 were retrospectively analysed. Indication for TURP followed current EAU Guidelines^1^. A detailed medical and sexual history was collected for every patient. Health-significant comorbidities were scored with the Charlson Comorbidity Index (CCI)^18^. We used the International Classification of Diseases, 9th revision. For the specific purpose of the analysis, CCI was categorised as 0 or ≥1. CCI was calculated considering patients reported health comorbidities such as diabetes mellitus, liver and kidney diseases, cardiovascular and neurological disorders as well as malignancies and AIDS^18^. Measured body mass index (BMI) was considered for each patient. Demographic information, patient factors and perioperative data were collected.

Demographic information included patient age at the time of the procedure, marital status, educational status and insurance type. The presence of psychiatric disorders in the cohort was assessed through patients’ reported history of any psychiatric or depressive disease and/or current or previous antipsychotic/antidepressive medications. Distance travelled for treatment was calculated using the distance from a patient’s home zip code to the hospital. Preoperative catheterization (POC) rate and time of POC were also considered. Prostate Specific Antigen (PSA), prostate volume (PV) and urinary maximum flow rate (Qmax) were collected for every patient. Intraoperative data included surgical time and the weight of resected tissue.

Postoperative factors included catheterization time, length of hospital stay and all complications, as well as postoperative-related events (PREs) occurring within 30 days. Complications were analysed according the Clavien-Dindo classification^19^. Patients received the histologic report at an office visit 15 days after surgery. Patients with histologic reports suggestive of incidental prostate cancer were excluded from the study (n = 4). Exclusion criteria were the presence of a known prostate or bladder cancer; neurogenic disorders; a history of bladder disease or other urologic conditions likely to affect micturition even after surgery.

Patient counselling and follow-up were standardized among the cohort. All patients received verbal preoperative counselling regarding the importance of follow-up to monitor for procedure-related outcomes and potential complications, such as urinary incontinence, urethral stenosis, prostate cancer development. PSA, urinary flow rate and post-voiding residual volume were scheduled 3-months after surgery. All patients received an appointment for follow-up before discharge after surgery. Patients were included if they were at least 60 days from their first scheduled follow-up appointment and were considered lost to follow-up if they were at least 30 days from their first scheduled appointment after surgery. Phone calls were used to investigate the rate of, and reason for being, LTF.

The primary endpoint of the study was to assess the proportion of patients who did not come for their follow-up assessment (namely, LTF patients). We also evaluated potential factors associated with patient nonadherence to prescribed follow-up after TURP.

Data collection was carried out following the principles outlined in the Declaration of Helsinki; after approval of the IRCCS Fondazione Ca’ Granda – Ospedale Maggiore Policlinico Ethical Committee, all patients signed an informed consent agreeing to supply their own anonymous data for this and future studies.

Data are presented as means (SD; ranges). The statistical significance of differences in means and proportions was tested with the one-way analysis of variance (ANOVA) and Pearson chi-square test, respectively. A 95% confidence interval (95% CI) was estimated for the association of categorical parameters. Exploratory analyses were initially applied to all variables; variables were retained for analysis when deemed clinically significant to the results. Descriptive statistics were used to assess potential differences in terms of clinical parameters and perioperative characteristics between LTF men and patients who attended the scheduled visit (namely, non-LTF). Univariable logistic regression (UVA) was performed to assess the impact of several variables on follow-up status. Logistic regression multivariable analysis (MVA) tested potential predictors (e.g. age, CCI, educational and marital status, distance travelled and PREs) of being LTF in our cohort of patients. Statistical analyses were performed using SPSS statistical software, v 13.0 (IBM Cor., Armonk, NY, USA). All tests were two sided, with a significance level set at 0.05.

### Data Availability Statement

All relevant data are within the paper and its Supporting Information files.

## Electronic supplementary material

Dataset 1
